# Trends and prescribing patterns of antimigraine medicines in nine major cities in China from 2018 to 2022: a retrospective prescription analysis

**DOI:** 10.1186/s10194-024-01775-6

**Published:** 2024-04-23

**Authors:** Jing Huang, Xinwei Wang, Yiyi Jin, Guodong Lou, Zhenwei Yu

**Affiliations:** 1 Department of Pharmacy, The First Affiliated Hospital of Ningbo University, Ningbo, 315010 China; 2 Department of Pharmacy, Sir Run Run Shaw Hospital, Zhejiang University School of Medicine, 3rd East Qingchun Road, Hangzhou, Zhejiang Province China

**Keywords:** Migraine, Headache, Prescribing patterns, NSAID, Opioid, Triptan, Medicine overuse

## Abstract

**Background:**

The objective of this study was to investigate the trends and prescribing patterns of antimigraine medicines in China.

**Methods:**

The prescription data of outpatients diagnosed with migraine between 2018 and 2022 were extracted from the Hospital Prescription Analysis Cooperative Project of China. The demographic characteristics of migraine patients, prescription trends, and corresponding expenditures on antimigraine medicines were analyzed. We also investigated prescribing patterns of combination therapy and medicine overuse.

**Results:**

A total of 32,246 outpatients who were diagnosed with migraine at 103 hospitals were included in this study. There were no significant trend changes in total outpatient visits, migraine prescriptions, or corresponding expenditures during the study period. Of the patients who were prescribed therapeutic medicines, 70.23% received analgesics, and 26.41% received migraine-specific agents. Nonsteroidal anti-inflammatory drugs (NSAIDs; 28.03%), caffeine-containing agents (22.15%), and opioids (16.00%) were the most commonly prescribed analgesics, with corresponding cost proportions of 11.35%, 4.08%, and 19.61%, respectively. Oral triptans (26.12%) were the most commonly prescribed migraine-specific agents and accounted for 62.21% of the total therapeutic expenditures. The proportion of patients receiving analgesic prescriptions increased from 65.25% in 2018 to 75.68% in 2022, and the proportion of patients receiving concomitant triptans decreased from 29.54% in 2018 to 21.55% in 2022 (both *P <  0.001*). The most frequently prescribed preventive medication classes were calcium channel blockers (CCBs; 51.59%), followed by antidepressants (20.59%) and anticonvulsants (15.82%), which accounted for 21.90%, 34.18%, and 24.15%, respectively, of the total preventive expenditures. Flunarizine (51.41%) was the most commonly prescribed preventive drug. Flupentixol/melitracen (7.53%) was the most commonly prescribed antidepressant. The most commonly prescribed anticonvulsant was topiramate (9.33%), which increased from 6.26% to 12.75% (both *P <  0.001*). A total of 3.88% of the patients received combined therapy for acute migraine treatment, and 18.63% received combined therapy for prevention. The prescriptions for 69.21% of opioids, 38.53% of caffeine-containing agents, 26.61% of NSAIDs, 13.97% of acetaminophen, and 6.03% of triptans were considered written medicine overuse.

**Conclusions:**

Migraine treatment gradually converges toward evidence-based and guideline-recommended treatment. Attention should be given to opioid prescribing, weak evidence-based antidepressant use, and medication overuse in migraine treatment.

**Supplementary Information:**

The online version contains supplementary material available at 10.1186/s10194-024-01775-6.

## Background

Migraine is a common neurological disorder that is commonly comorbid with a range of conditions and diseases, including depression, anxiety, chronic pain, and epilepsy [1]. It affects more than 1 billion people worldwide [2]. China is a country with a large number of migraine sufferers, and the one-year incidence of migraine is reported to be approximately 9% to 14% [3]. According to the 2016 Global Burden of Diseases (GBD) study, migraine is the second most debilitating disease concerning years of life lived with disability [4]. It has a range of negative effects not only on affected people but also on their families, employers, and society [5]. There is an urgent need for better treatment and management of migraine patients.

Antimigraine medicines are an essential component of optimal migraine treatment. The use of antimigraine medicines varies among countries and regions [6–8]. It is influenced by drug availability, drug profiles, physician and patient preferences, reimbursement and drug regulation policies, and other factors [2, 9]. Information on the prescribing patterns and treatment costs of migraine in China is limited. There are also concerns about whether antimigraine prescriptions are always justified, such as whether the drug choice is appropriate, whether drug combinations are necessary, and whether medicine overuse prescriptions exist. Thus, understanding the current situation regarding antimigraine medication use is critical, as it can provide a basis for improved antimigraine medication management. This study aimed to evaluate the trends and patterns of the use of antimigraine medicines from 2018 to 2022 in nine major cities in China.

## Methods

### Study design and data source

We performed a database-based, cross-sectional study using data from 2018 to 2022. The migraine prescription data were obtained from the Hospital Prescription Analysis Cooperative Project of China, which is widely used in pharmacoepidemiology studies [10–12]. The database contained prescription information on 40 randomized sampling days per year (10 sampling days each quarter) from the participating hospitals. Each prescription contained information on the prescription code, hospital code, prescription date, clinical department, gender and age of the patient, patient’s diagnosis, and detailed information on the dispensed drug (name, pharmacological classification, formulation, size, dose, frequency, route of administration and cost). This study was approved by the Ethics Committee of Sir Run Run Shaw Hospital, College of Medicine, Zhejiang University. Informed consent was waived due to the retrospective nature of the study.

### Study population and inclusion criteria for migraine prescriptions

Outpatients of all ages who received treatment for a diagnosis of migraine belonging to the *International Statistical Classification of Diseases and Related Health Problems (ICD) revision 10* (*ICD-10*) code (*G43.xxx*), were included in the study. Outpatient prescriptions that met the following criteria were extracted from the database: (1) prescriptions from hospitals located in nine major areas of China (Beijing, Chengdu, Guangzhou, Haerbin, Hangzhou, Shanghai, Shenyang, Tianjin, Zhengzhou); (2) prescriptions from hospitals that participated in the program continuously from 2018 to 2022; and (3) prescriptions for antimigraine medications administered by the oral, injectable, nasal, or anal route of administration were included.

### Drug classification and prescription screening

According to the migraine treatment guidelines and the current condition of migraine treatment in China, antimigraine medications were categorized into therapeutic and preventive medications [13–15]. Therapeutic medications include NSAIDs, caffeine-containing agents, opioids, acetaminophen, Chinese patent medicines (CPMs), triptans, ergotamine, and antiemetics. Preventive medications include calcium channel blockers (CCBs), antidepressants, anticonvulsants, calcium channel modulators (CCMs), beta-blockers, and other recommended agents. CPMs are herbal extracts or ingredients with blood-activating and analgesic properties or are approved for headache treatment in China.

The extracted prescriptions were categorized by medicines, and those that met the above medicine categories were included in the study and classified into the respective therapeutic or preventive medicine classes based on their pharmacological effects. Medicines that were not in the above medicine categories were considered non-treatment medicines for migraine and were not included in the study. Prescriptions for the included medicine were excluded if they were used for disease treatment purposes other than migraine: (1) ibuprofen, loxoprofen, indomethacin, acetaminophen, and caffeine-containing agent prescriptions with a first diagnosis of fever; or (2) aspirin prescriptions with a diagnosis of coronary heart disease, angina, stroke, or transient ischemic attack for antiplatelet purpose at a daily dose of < 300 mg. All antimigraine medications involved in this study within the therapeutic or preventive medication categories are listed in Supplementary Table 1.

### Prescribing patterns and overuse definition

Prescribing patterns were analyzed as monotherapy or combined therapy. Monotherapy was defined as only one antimigraine medication written on a single prescription, and combined therapy was defined as two or more antimigraine medications written on a single prescription.

According to the International Classification of Headache Disorders, Third Edition (ICHD-3), a prescription of ergots, triptans, or opioids for a course of ≥10 days or nonopioid analgesics (acetaminophen and NSAIDs) for ≥15 days was considered an overuse prescription in the present study [16]. The prescription course was calculated using the following equation, and the number of medicine overuse prescriptions was calculated.

1 $$\textrm{Prescription}\ \textrm{course}=\frac{dose\ per\ tablet\ast size\ast quantity}{single\ dose\ast frequency}$$

## Data analysis

The demographic characteristics of patients diagnosed with migraine were evaluated through outpatient visits, regardless of whether the patients were first diagnosed or renewed. The trends of patients diagnosed with migraine were further stratified by age and sex. Overall trends of antimigraine medication use in prescriptions and costs were described over the five-year observation period. The yearly prescriptions and the proportions of annual and total prescriptions of different classes and specific antimigraine medications were calculated. The total cost, therapeutic medicine cost, and preventative medicine cost were calculated by summing all the costs of antimigraine medicines, summing all the costs of therapeutic medicines, and summing all the costs of preventative medicines, respectively. The rank-sum test was applied to determine the statistical significance of overall trends for outpatient visits and expenditures. The statistical significance of the trends in the identified values was tested by the Mann–Kendal test and the trends in proportions were assessed by the Cochran–Armitage trend test. The prescription data were processed using Microsoft Office Excel V.2016 (Microsoft Corp., Redmond, WA, USA). All of the statistical analyses were conducted using R V.4.3.2 (http://www.R-project.org).

## Results

### Demographic characteristics of outpatients with migraine and trends in overall use

**Fig. 1:**
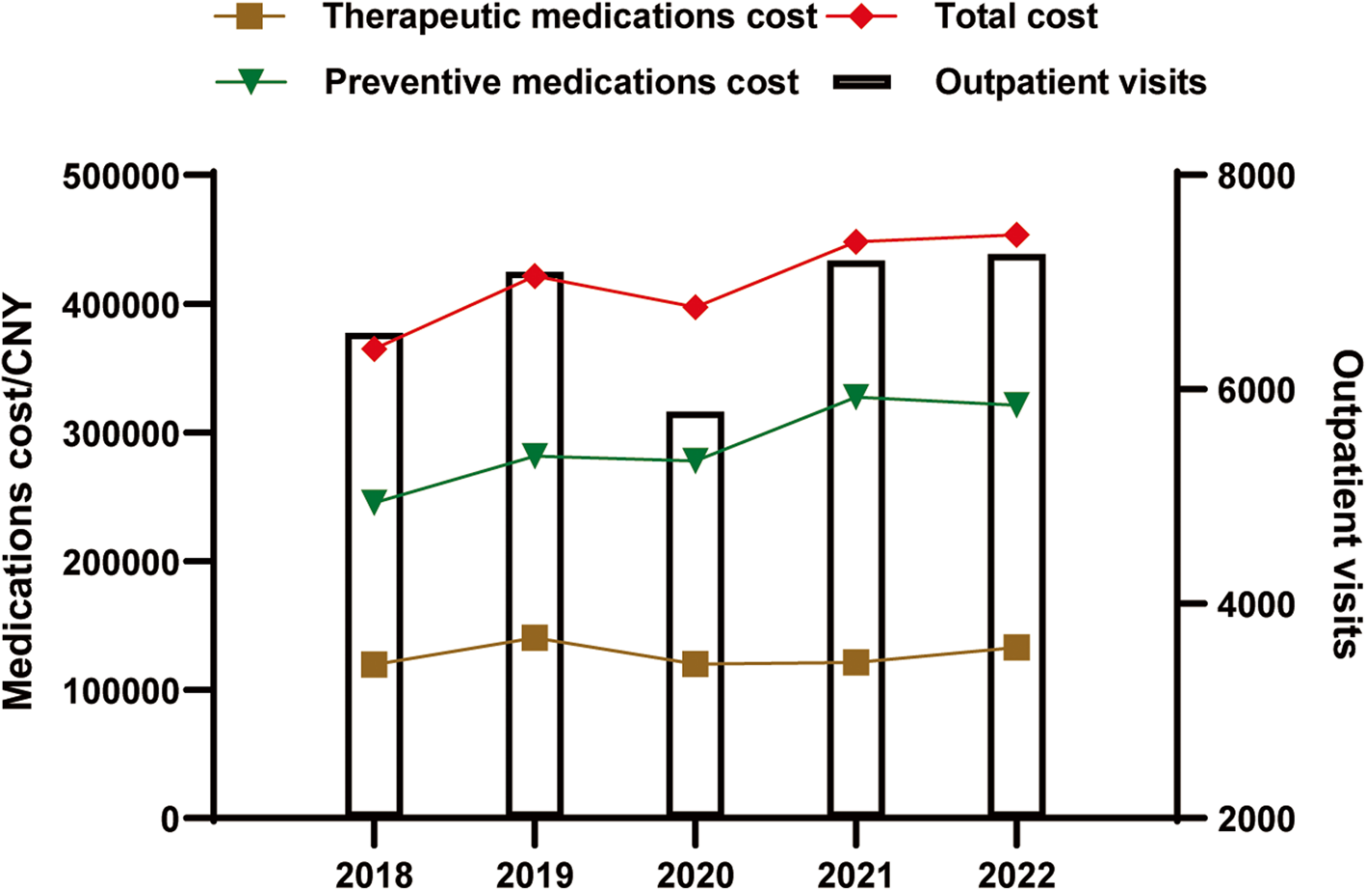
Trends in outpatient visits and cost of antimigraine medications from 2018 to 2022

The data of a total of 32,246 patients who were diagnosed with migraine in 103 hospitals in the nine cities were extracted. The demographic characteristics of the total study population and the population stratified by age group and sex between 2018 and 2022 are shown in Table 1. Migraine visits were concentrated in those aged 30–39 years (21.66%), 40–49 years (19.31%), and 50–59 years (18.59%). The proportion of outpatients aged 30–39 years continuously increased (*P <  0.001*), while that of patients aged ≥70 years decreased dramatically (*P <  0.001*). There were approximately 2.3 times more female migraine patients than male migraine patients, with the proportion of female patients showing a continuing upward trend from 64.68% in 2018 to 69.75% in 2022 (*P <  0.001*). The most frequently visited department for migraine patients was neurology (61.13%), followed by internal medicine (5.84%) and the emergency department (5.42%). As shown in Fig. 1, there were no significant trend changes in total outpatient visits, therapeutic or preventative prescriptions, or corresponding expenditures during the study period (both *P > 0.05*).

**Table 1:** Demographic characteristics of outpatients diagnosed with migraine

	2018	2019	2020	2021	2022	Total	*P*_*1*_	*P*_*2*_
Outpatient visits	6217	6766	5418	6748	7097	32,246	0.462	–
Age (years)								
<30	1036 (16.66)	1037 (15.33)	896 (16.54)	1204 (17.84)	1244 (17.53)	5417 (16.80)	0.221	0.003
30–39	1191 (19.16)	1340 (19.80)	1224 (22.59)	1601 (23.73)	1630 (22.97)	6986 (21.66)	0.086	< 0.001
40–49	1295 (20.83)	1290 (19.07)	1021 (18.84)	1275 (18.89)	1345 (18.95)	6226 (19.31)	1.000	0.014
50–59	1175 (18.90)	1318 (19.48)	1016 (18.75)	1202 (17.81)	1285 (18.11)	5996 (18.59)	0.807	0.029
60–69	840 (13.51)	1027 (15.18)	719 (13.27)	848 (12.57)	905 (12.75)	4339 (13.46)	0.807	0.001
≥ 70	647 (10.41)	709 (10.48)	504 (9.30)	564 (8.36)	619 (8.72)	3043 (9.44)	0.807	< 0.001
sex								
male	1912 (30.75)	2006 (29.65)	1603 (29.59)	1892 (28.04)	1890 (26.63)	9303 (28.85)	0.462	< 0.001
female	4021 (64.68)	4511 (66.67)	3575 (65.98)	4551 (67.44)	4950 (69.75)	21,608 (67.01)	0.221	< 0.001

*P*_*1*_, *P value* for the trend in the number of outpatient visits, assessed by the Mann–Kendall trend test; *P*_*2*_, *P value* for the trend in the proportion of outpatient visits, assessed by the Cochran–Armitage trend test. The number of outpatient visits for each year is the denominator of the data in that column

### Prescriptions and expenditures of therapeutic antimigraine medications

**Fig. 2:**
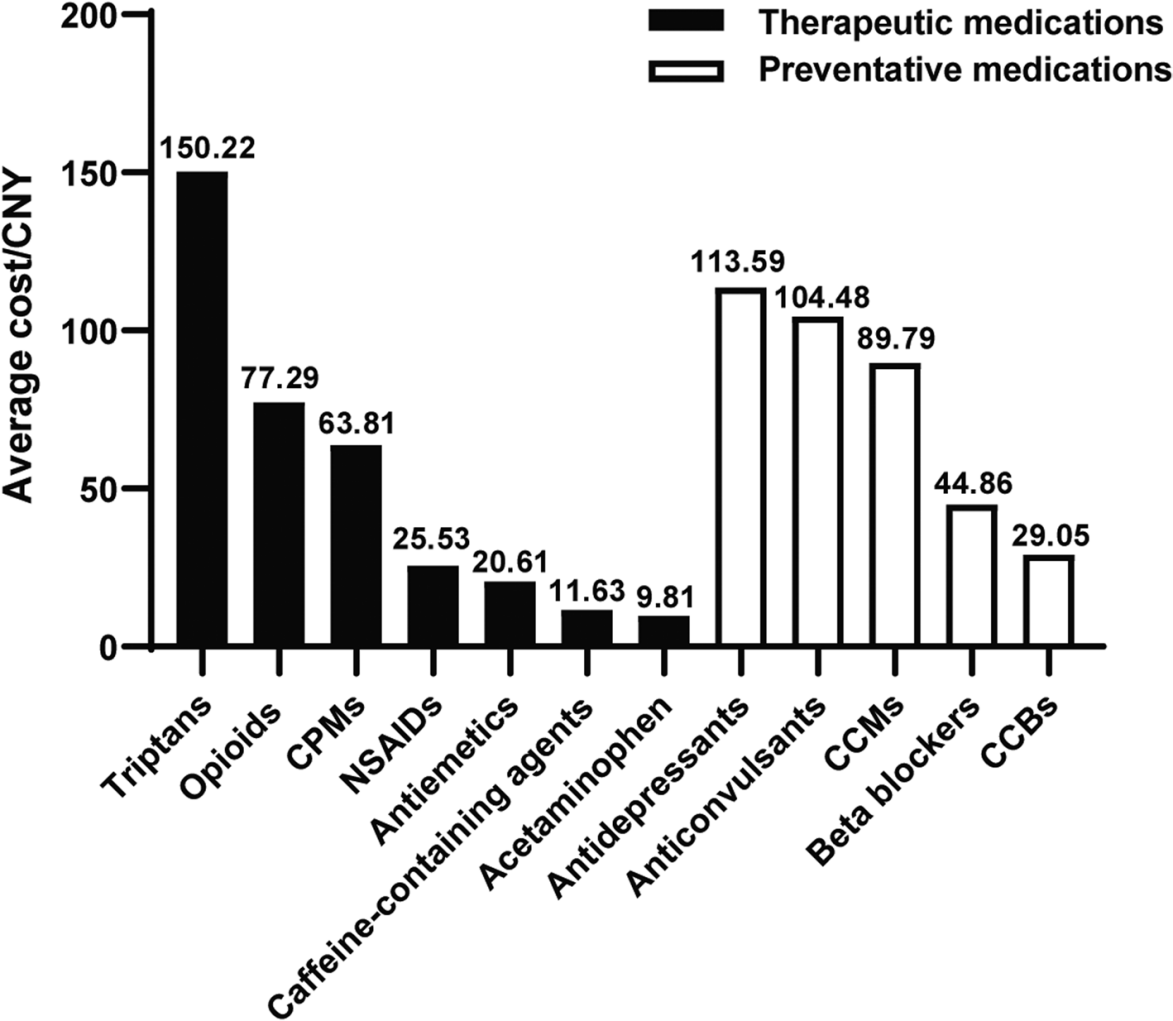
Average cost per prescription of the different antimigraine medications. CPMs, Chinese patent medicines; NSAIDs, nonsteroidal anti-inflammatory drugs; CCMs, calcium channel modulators; CCBs, calcium channel blockers; CNY, Chinese Yuan

The therapeutic prescriptions and expenditures for antimigraine medications were examined, and the results are shown in Tables 2 and 3. Analgesics were favored over migraine-specific agents, with a 5-year average proportion of 70.23% for analgesic prescriptions and 26.41% for migraine-specific prescriptions; the remaining 3.36% were antiemetic prescriptions. NSAIDs (28.03%), caffeine-containing agents (22.15%), and opioids (16.00%) were the most commonly prescribed analgesics, with corresponding cost proportions of 11.35%, 4.08%, and 19.61%, respectively. Oral triptans (26.12%) were the most commonly prescribed migraine-specific agents and accounted for 62.21% of the total therapeutic expenditures. The most commonly used NSAIDs were ibuprofen (10.23%), loxoprofen (4.47%), and celecoxib (3.60%). The most commonly used opioids were codeine/ibuprofen (10.27%) and dihydrocodeine/acetaminophen (2.14%). Rizatriptan (17.74%) and zolmitriptan (8.09%) were the most commonly prescribed triptans. The highest-ranked average cost per prescription was 150.22 CNY for triptans, 77.29 CNY for opioids, and 63.81 CNY for CPMs (Fig. 2). In terms of trends in therapeutic drug prescription and cost, the proportion of patients receiving analgesic prescriptions increased from 65.25% in 2018 to 75.68% in 2022, the proportion of patients receiving mainly NSAIDs increased from 24.50% to 34.38%, the proportion receiving caffeine-containing agents increased from 19.77% to 24.85%, and the corresponding drug expenditures increased accordingly (both *P <  0.001*). Moreover, triptan use decreased from 29.54% in 2018 to 21.55% in 2022 (*P <  0.001*). The proportions of patients who received opioid (*P = 0.004*) or CPM (*P <  0.001*) prescriptions also declined.

**Table 2:** Trends of therapeutic drugs prescribed in outpatients with migraine between 2018 and 2022

	2018	2019	2020	2021	2022	Total	*P*_*1*_	*P*_*2*_
**Total therapeutic drugs**	1882	2096	1699	2010	2237	9924	0.133	–
**Analgesics**	1228 (65.25)	1404 (66.98)	1218 (71.69)	1427 (71.00)	1693 (75.68)	6970 (70.23)	0.221	< 0.001
NSAIDs	461 (24.50)	477 (22.76)	514 (30.25)	561 (27.91)	769 (34.38)	2782 (28.03)	0.028	< 0.001
Ibuprofen	161 (8.55)	158 (7.54)	181 (10.65)	189 (9.40)	321 (14.35)	1010 (10.18)	0.086	< 0.001
loxoprofen	67 (3.56)	64 (3.05)	86 (5.06)	69 (3.43)	107 (4.78)	393 (3.96)	0.221	< 0.001
Celecoxib	45 (2.39)	63 (3.01)	70 (4.12)	87 (4.33)	92 (4.11)	357 (3.60)	0.028	< 0.001
Caffeine-containing agents	372 (19.77)	422 (20.13)	379 (22.31)	469 (23.33)	556 (24.85)	2198 (22.15)	0.086	< 0.001
Opioids	298 (15.83)	388 (18.51)	267 (15.72)	338 (16.82)	297 (13.28)	1588 (16.00)	0.807	0.004
Codeine/Ibuprofen	192 (10.20)	236 (11.26)	174 (10.24)	217 (10.80)	200 (8.94)	1019 (10.27)	1.000	0.123
Dihydrocodeine/acetaminophen	30 (1.59)	67 (3.20)	23 (1.35)	49 (2.44)	43 (1.92)	212 (2.14)	1.000	0.797
Acetaminophen	38 (2.02)	46 (2.19)	25 (1.47)	29 (1.44)	46 (2.06)	184 (1.85)	1.000	0.516
CPMs	59 (3.13)	71 (3.39)	33 (1.94)	30 (1.49)	25 (1.12)	218 (2.20)	0.086	< 0.001
**Migraine-specific agents**	585 (31.08)	604 (28.82)	424 (24.96)	526 (26.17)	482 (21.55)	2621 (26.41)	0.462	< 0.001
Triptans	556 (29.54)	604 (28.82)	424 (24.96)	526 (26.17)	482 (21.55)	2592 (26.12)	0.462	< 0.001
Rizatriptan	331 (17.59)	414 (19.75)	288 (16.95)	379 (18.86)	349 (15.60)	1761 (17.74)	1.000	0.045
zolmitriptan	197 (10.47)	190 (9.06)	136 (8.00)	147 (7.31)	133 (5.95)	803 (8.09)	0.086	< 0.001
Ergotamine	29 (1.54)	0 (0.00)	0 (0.00)	0 (0.00)	0 (0.00)	29 (0.29)	0.289	< 0.001
**Antiemetics**	69 (3.67)	88 (4.20)	57 (3.35)	57 (2.84)	62 (2.77)	333 (3.36)	0.613	0.011

*NSAIDs* nonsteroidal anti-inflammatory drugs, *CPMs* Chinese patent medicines. *P*_*1*_*, P value* for the trend in number of prescriptions, assessed by the Mann-Kendall trend test; *P*_*2*_*, P value* for the trend in proportion of prescriptions, assessed by the Cochran-Armitage trend test. The number of total therapeutic drugs for each year is the denominator of the data in that column

**Table 3:** Expenditure of therapeutic drugs dispensed between 2018 and 2022

	2018	2019	2020	2021	2022	Total	*P*_*1*_	*P*_*2*_
**Total therapeutic drugs**	118,297.91	138,338.85	118,115.51	119,952.31	131,219.4	625,923.98	0.807	–
**Analgesics**	41,315.1 (34.92)	49,248.39 (35.60)	45,255.99 (38.32)	52,110.56 (43.44)	47,107.48 (35.90)	235,037.52 (37.55)	0.462	< 0.001
NSAIDs	10,928.15 (9.24)	12,455.82 (9.00)	15,876.91 (13.44)	12,799.12 (10.67)	18,969.65 (14.46)	71,029.65 (11.35)	0.086	< 0.001
Ibuprofen	2200.35 (1.86)	2679.09 (1.94)	2720.93 (2.30)	1446.24 (1.21)	3051.65 (2.33)	12,098.26 (1.93)	0.462	0.035
loxoprofen	2251.35 (1.90)	2039.09 (1.47)	2834.1 (2.40)	1635.78 (1.36)	1522.87 (1.16)	10,283.19 (1.64)	0.221	< 0.001
Celecoxib	2221.94 (1.88)	3452.58 (2.50)	5206.86 (4.41)	3096.49 (2.58)	3160.50 (2.41)	17,138.37 (2.74)	0.807	< 0.001
Caffeine-containing agents	4202.91 (3.55)	4927.46 (3.56)	4685.18 (3.97)	5944.38 (4.96)	5793.78 (4.42)	25,553.71 (4.08)	0.462	< 0.001
Opioids	22,237.89 (18.80)	26,689.2 (19.29)	22,059.9 (18.68)	30,866.94 (25.73)	20,883.71 (15.92)	122,737.64 (19.61)	0.462	0.987
Codeine/Ibuprofen	13,775.59 (11.64)	15,979.8 (11.55)	11,367.54 (9.62)	14,084.68 (11.74)	12,381.48 (9.44)	67,589.09 (10.80)	0.807	< 0.001
Dihydrocodeine/acetaminophen	1888.4 (1.60)	3458.74 (2.50)	1470.24 (1.24)	3073.32 (2.56)	2935.92 (2.24)	12,826.62 (2.05)	1.000	< 0.001
Acetaminophen	357.25 (0.30)	530.52 (0.38)	291.16 (0.25)	225.28 (0.19)	401.30 (0.31)	1805.51 (0.29)	0.807	< 0.001
CPMs	3588.9 (3.03)	4645.39 (3.36)	2342.84 (1.98)	2274.84 (1.90)	1059.04 (0.81)	13,911.01 (2.22)	0.086	< 0.001
**Migraine-specific agents**	76,982.81 (65.08)	89,090.46 (64.40)	72,859.52 (61.68)	67,841.75 (56.56)	84,111.92 (64.10)	390,886.46 (62.45)	0.807	< 0.001
Triptans	75,457.21 (63.79)	89,090.46 (64.40)	72,859.52 (61.68)	67,841.75 (56.56)	84,111.92 (64.10)	389,360.86 (62.21)	0.807	< 0.001
Rizatriptan	51,573.48 (43.60)	67,463.94 (48.77)	57,988.95 (49.10)	51,724.57 (43.12)	64,657.86 (49.27)	293,408.8 (46.88)	0.807	< 0.001
zolmitriptan	22,599.21 (19.10)	21,626.52 (15.63)	14,870.57 (12.59)	16,117.18 (13.44)	19,454.06 (14.83)	94,667.54 (15.12)	0.462	< 0.001
Ergotamine	1525.6 (1.29)	0 (0.00)	0 (0.00)	0 (0.00)	0 (0.00)	1525.6 (0.24)	0.289	< 0.001
**Antiemetics**	1187.64 (1.00)	1814.57 (1.31)	1536.61 (1.30)	954.12 (0.80)	1368.71 (1.04)	6861.65 (1.10)	0.807	< 0.001

*NSAIDs* nonsteroidal anti-inflammatory drugs, *CPMs* Chinese patent medicines. *P*_*1*_*, P value* for the trend in number of prescriptions, assessed by the Mann-Kendall trend test; *P*_*2*_*, P value* for the trend in the proportion of prescriptions, assessed by the Cochran-Armitage trend test. The number of total therapeutic drugs for each year is the denominator of the data in that column

### Prescriptions and expenditures of preventive antimigraine medications

The preventive prescriptions and expenditures for antimigraine medications were examined, and the results are shown in Tables 4 and 5. The most frequently prescribed preventive medication class was CCBs (51.59%), followed by antidepressants (20.59%), anticonvulsants (15.82%), beta-blockers (6.91%), and CCMs (4.61%), with corresponding expenditures of 21.90%, 34.18%, 24.15%, 4.53%, and 6.05%, respectively. The average cost per prescription was 113.59 CNY for antidepressants, 104.48 CNY for anticonvulsants, 89.79 CNY for CCMs, 44.86 CNY for beta-blockers, and 29.05 CNY for CCBs (Fig. 2). Flunarizine was the most commonly prescribed preventive drug, accounting for almost half of the prescriptions (51.41%). A continuous decline in flunarizine use was observed from 53.12% in 2018 to 47.52% in 2022, consistent with the decline in expenditure from 24.17% to 17.79% (both *P <  0.001*). Antidepressant use also decreased from 21.26% in 2018 to 18.41% in 2022, while the corresponding expenditure decreased significantly, from 41.38% to 23.97%. In particular, the prescription proportion of the most commonly used antidepressant was flupentixol/melitracen, with the proportion of prescriptions decreasing from 8.24% to 5.50%. In contrast, topiramate prescriptions increased from 250 in 2018 to 563 in 2022, with a dramatic increase in the proportion of prescriptions from 6.26% to 12.75%, and the corresponding expenditure increased from 24,308.78 CNY to 59,100.48 CNY, with an increase in the proportion of expenditures from 9.91% to 18.42% (both *P <  0.001*). The number and proportion of pregabalin prescriptions increased significantly (both *P <  0.001*), as did the proportion of pregabalin expenditures (both *P <  0.001*).

**Table 4:** Trends of preventive drugs prescribed in outpatients with migraine between 2018 and 2022

	2018	2019	2020	2021	2022	Total	*P*_*1*_	*P*_*2*_
**Total preventative drugs**	3993	4500	3628	4688	4415	21,224	0.807	–
CCB	2160 (54.09)	2380 (52.89)	1910 (52.65)	2402 (51.24)	2098 (47.52)	10,950 (51.59)	1.000	< 0.001
Flunarizine	2121 (53.12)	2380 (52.89)	1910 (52.65)	2402 (51.24)	2098 (47.52)	10,911 (51.41)	1.000	< 0.001
Antidepressants	849 (21.26)	997 (22.16)	686 (18.91)	1025 (21.86)	813 (18.41)	4370 (20.59)	1.000	0.002
Flupentixol/melitracen	329 (8.24)	416 (9.24)	251 (6.92)	360 (7.68)	243 (5.50)	1599 (7.53)	0.462	< 0.001
Amitriptyline	123 (3.08)	110 (2.44)	88 (2.43)	182 (3.88)	124 (2.81)	627 (2.95)	0.807	0.214
Escitalopram	78 (1.95)	125 (2.78)	77 (2.12)	136 (2.90)	81 (1.83)	497 (2.34)	0.807	0.819
Duloxetine	82 (2.05)	90 (2.00)	66 (1.82)	98 (2.09)	95 (2.15)	431 (2.03)	0.462	0.655
Anticonvulsants	530 (13.27)	585 (13.00)	599 (16.51)	764 (16.30)	879 (19.91)	3357 (15.82)	0.028	< 0.001
Topiramate	250 (6.26)	315 (7.00)	377 (10.39)	476 (10.15)	563 (12.75)	1981 (9.33)	0.028	< 0.001
Valproate	201 (5.03)	212 (4.71)	166 (4.58)	214 (4.56)	221 (5.01)	1014 (4.78)	0.221	0.869
CCMs	157 (3.93)	193 (4.29)	160 (4.41)	191 (4.07)	277 (6.27)	978 (4.61)	0.221	< 0.001
Gabapentin	119 (2.98)	154 (3.42)	100 (2.76)	111 (2.37)	124 (2.81)	608 (2.86)	1.000	0.070
Pregabalin	38 (0.95)	39 (0.87)	60 (1.65)	80 (1.71)	153 (3.47)	370 (1.74)	0.028	< 0.001
Beta blockers	282 (7.06)	327 (7.27)	243 (6.70)	290 (6.19)	324 (7.34)	1466 (6.91)	0.807	0.643
Metoprolol	158 (3.96)	214 (4.76)	136 (3.75)	176 (3.75)	196 (4.44)	880 (4.15)	0.807	0.908
Propranolol	73 (1.83)	68 (1.51)	60 (1.65)	56 (1.19)	58 (1.31)	315 (1.48)	0.086	0.022
Other agents	23 (0.58)	18 (0.40)	30 (0.83)	16 (0.34)	24 (0.54)	103 (0.49)	1.000	0.718

*CCBs* Calcium channel blockers, *CCMs* calcium channel modulators. *P*_*1*_*, P value* for trend in number of prescriptions, assessed by the Mann-Kendall trend test; *P*_*2*_*, P value* for trend in proportion of prescriptions, assessed by the Cochran-Armitage trend test. The number of total preventative drugs for each year is the denominator of the data in that column

**Table 5:** Expenditure of preventive drugs dispensed between 2018 and 2022

	2018	2019	2020	2021	2022	Total	*P*_*1*_	*P*_*2*_
**Total preventative drugs**	245,363.86	281,422.20	277,575.91	327,095.25	320,843.99	1,452,301.21	0.221	–
CCBs	60,487.77 (24.65)	72,853.12 (25.89)	57,446.59 (20.70)	70,194.61 (21.46)	57,062.85 (17.79)	318,044.94 (21.90)	0.462	< 0.001
Flunarizine	59,299.05 (24.17)	72,853.12 (25.89)	57,446.59 (20.70)	70,194.61 (21.46)	57,062.85 (17.79)	316,856.22 (21.82)	0.462	< 0.001
Antidepressants	101,538.71 (41.38)	117,488.23 (41.75)	92,735.89 (33.41)	107,734.09 (32.94)	76,892.91 (23.97)	496,389.83 (34.18)	0.462	< 0.001
Flupentixol/melitracen	23,560.92 (9.60)	33,441.56 (11.88)	22,165.50 (7.99)	30,767.90 (9.41)	20,623.98 (6.43)	130,559.86 (8.99)	0.462	< 0.001
Amitriptyline	1395.06 (0.57)	1405.31 (0.50)	1602.08 (0.58)	2759.29 (0.84)	1232.31 (0.38)	8394.05 (0.58)	0.807	0.398
Escitalopram	35,524.44 (14.48)	36,671.57 (13.03)	14,199.23 (5.12)	25,490.65 (7.79)	16,238.36 (5.06)	128,124.25 (8.82)	0.462	< 0.001
Anticonvulsants	45,860.12 (18.69)	50,422.43 (17.92)	58,794.05 (21.18)	93,473.04 (28.58)	102,190.54 (31.85)	350,740.18 (24.15)	0.028	< 0.001
Topiramate	24,308.78 (9.91)	30,299.16 (10.77)	40,037.64 (14.42)	54,532.44 (16.67)	59,100.48 (18.42)	208,278.50 (14.34)	0.028	< 0.001
Valproate	11,673.63 (4.76)	11,790.05 (4.19)	10,083.37 (3.63)	18,657.67 (5.70)	17,593.05 (5.48)	69,797.77 (4.81)	0.462	< 0.001
CCMs	12,529.74 (5.11)	15,122.32 (5.37)	20,802.53 (7.49)	16,278.36 (4.98)	23,079.52 (7.19)	87,812.47 (6.05)	0.086	< 0.001
Gabapentin	6346.28 (2.59)	7883.74 (2.80)	5711.74 (2.06)	3468.40 (1.06)	1703.20 (0.53)	25,113.36 (1.73)	0.086	< 0.001
Pregabalin	6183.46 (2.52)	7238.58 (2.57)	15,090.79 (5.44)	12,809.96 (3.92)	21,376.32 (6.66)	62,699.11 (4.32)	0.086	< 0.001
Beta blockers	9643.72 (3.93)	12,800.38 (4.55)	12,341.97 (4.45)	15,395.31 (4.71)	15,588.51 (4.86)	65,769.89 (4.53)	0.086	< 0.001
Metoprolol	4679.27 (1.91)	8064.44 (2.87)	6384.54 (2.30)	8162.081 (2.50)	8946.85 (2.79)	36,237.18 (2.50)	0.086	< 0.001
Propranolol	1498.06 (0.61)	1228.10 (0.44)	1127.36 (0.41)	1282.85 (0.39)	1268.64 (0.40)	6405.01 (0.44)	0.807	< 0.001
Other agents	15,303.8 (6.24)	12,735.72 (4.53)	35,454.88 (12.77)	24,019.84 (7.34)	46,029.66 (14.35)	133,543.9 (9.20)	0.221	< 0.001

*CCBs* Calcium channel blockers, *CCMs* calcium channel modulators. *P*_*1*_*, P value* for the trend in number of prescriptions, assessed by the Mann-Kendall trend test; *P*_*2*_*, P value* for the trend in proportion of prescriptions, assessed by the Cochran-Armitage trend test. The number of total preventative drugs for each year is the denominator of the data in that column

### Prescribing patterns

**Fig. 3:**
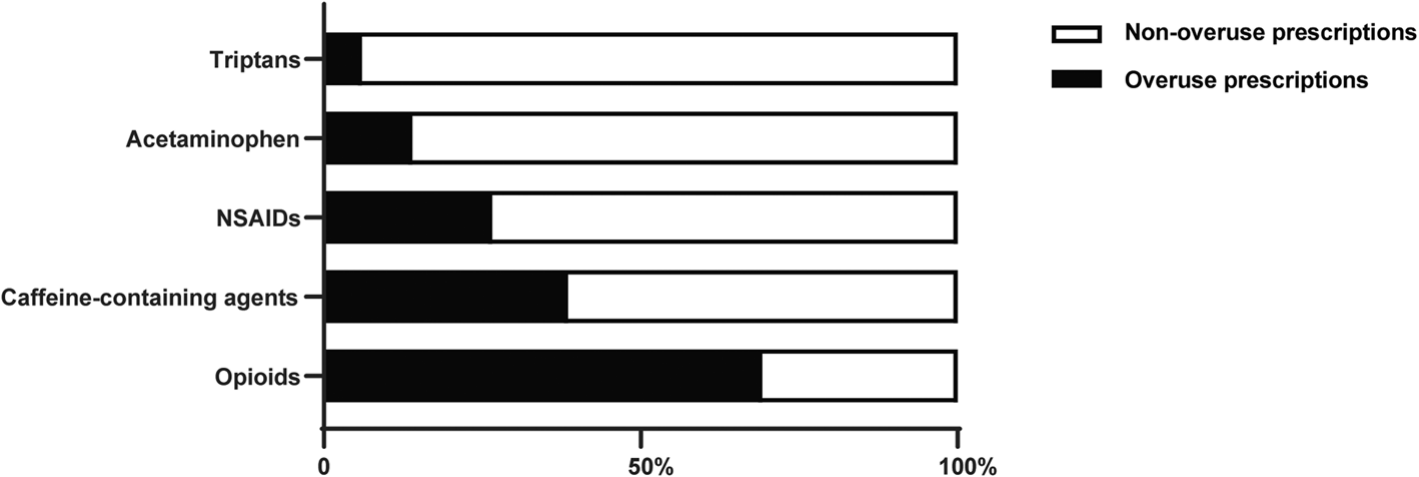
Proportions of prescriptions with medicine overuse

We analyzed prescribing patterns for monotherapy/combination therapy and medicine overuse prescriptions. Monotherapy was more widely used than combined therapy for both therapeutic and preventive migraine treatment, with proportions of 96.12% versus 3.88% and 81.37% versus 18.63%, respectively (Table 6). The proportion of patients receiving combined preventive therapy increased from 17.38% in 2018 to 20.08% in 2022, and the proportion of patients receiving monotherapy decreased (both *P = 0.002*). The most frequently used combination for therapeutic migraine treatment was a triptan-based combination, which accounted for 63.58% of the combination prescriptions. The most frequently used combination for preventive migraine treatment was a flunarizine-based combination, which accounted for 51.83% of the combination prescriptions. As revealed in Fig. 3, 69.21% of opioid prescriptions, 38.53% of caffeine-containing agent prescriptions, 26.61% of NSAID prescriptions, 13.97% of acetaminophen prescriptions, and 6.03% of triptan prescriptions considered written medicine overuse.

**Table 6:** Trends of multidrug combinations prescribed for outpatients with migraine between 2018 and 2022

	2018	2019	2020	2021	2022	Total	*P*_*1*_	*P*_*2*_
Outpatients receiving therapeutic drugs*	1748	1916	1571	1867	2104	9206	–	–
Monotherapy	1688 (96.57)	1828 (95.41)	1504 (95.74)	1786 (95.66)	2043 (97.10)	8849 (96.12)	0.462	0.250
Combined therapy	60 (3.43)	88 (4.59)	67 (4.26)	81 (4.34)	61 (2.90)	357 (3.88)	1.000	0.250
Outpatients receiving preventive drugs	3337	3749	3009	3839	3555	17,494	–	–
Monotherapy	2757 (82.62)	3069 (81.86)	2460 (81.75)	3108 (80.96)	2841 (79.92)	14,235 (81.37)	0.807	0.002
Combined therapy	580 (17.38)	680 (18.13)	549 (18.24)	731 (19.04)	714 (20.08)	3259 (18.63)	0.462	0.002

^*^ Outpatients receiving antiemetics were not included. The number of outpatients receiving therapeutic drugs and the number of outpatients receiving preventive drugs for each year are the denominators of monotherapy and combined therapy in that column, respectively

## Discussion

This study assessed the trends and prescribing patterns of antimigraine medicines in nine major Chinese cities using a large anonymized database. Trend changes occurred in the prescriptions and expenditures for antimigraine medicine classes and certain medicines. The use of nonrecommended and weak evidence-based migraine medications and medication overuse prescriptions were also identified.

Analgesics were found to be the main medicines for acute migraine treatment. Acetaminophen, NSAIDs, and caffeine-containing agents were reported to be effective in randomized placebo-controlled trials of migraine therapy and are the recommended medications for acute migraine treatment according to Chinese and international guidelines [13, 15]. In our study, 50.18% of these three nonopioid analgesics consumed only 15.43% of the therapeutic drug costs. As a highly cost-effective treatment for migraine, these three nonopioid analgesics remain the first choice for patients with mild to moderate migraine in China, with caution given to adverse effects such as gastrointestinal upset and renal injury. Opioids accounted for 16% of prescriptions for migraine patients, even though the Chinese guidelines for migraine treatment warned that opioids should only be considered a medicine for severe headaches that fail to respond to other medications. Opioids are known to have no better efficacy than NSAIDs for treating migraine, and they are associated with higher rates of migraine recurrence and greater adverse effects, are potentially addictive, and are commonly implicated in medication overuse headache (MOH) [17, 18]. Previous studies in many countries and regions have concluded that prescribing opioids for migraine attacks is common [19, 20]. The 16% of opioid use suggests that opioid control remains inadequate, at least in migraine treatment. Although opioid use trended downward during the study period, more effective control of opioids is nonetheless needed. There were also 2.2% of CPM prescriptions, which were unique to China. Migraine is referred to *Shou Feng* in traditional Chinese medicine (TCM) term*.* Medicine treatment is based on clinical symptoms and signs, and CPMs with blood-activating and analgesic properties are frequently prescribed for the treatment of migraine. Given the lack of high-quality clinical evidence, CPMs are more often used as adjuncts to migraine treatment in combination with Western medicine.

Migraine-specific agents accounted for 26.41% of the therapeutic prescriptions. Ergotamines, which have unpredictable bioavailability and poor tolerability, are generally obsolete and have been replaced by triptans. The use of triptans, which are serotonin 1b/1d agonists, is supported by level A evidence, and triptans are recognized as effective drugs for acute migraine treatment by Chinese and international treatment guidelines [13, 14]. Triptans are reimbursed by China’s health insurance only for second-line treatment of acute migraine that has failed to respond to other analgesics, which limits their use in China. There were three triptans available in China during the study period; sumatriptan was seldom chosen, while rizatriptan constituted approximately 68% of triptan prescriptions, and zolmitriptan constituted 31%. Our data illustrated that 26.12% of triptan prescriptions generated 62.21% of therapeutic drug costs, and the average per prescription was 150.22 CNY, indicating that triptans are relatively expensive. As reported in other countries, high triptan costs generate a significant societal burden [21]. A previous study revealed that triptans accounted for only 3.3% of acute migraine therapeutic medications in China from 2016 to 2017 [22]. This is comparable to the 26.12% in our study. This may be related to the increased awareness and understanding of migraine-specific medicine use, and drug availability in these nine cities. Triptans are more accessible in major cities in China than in other cities and rural areas, which allows physicians and patients to choose more effective triptans. Despite a significant increase in the use of triptan over the previous study, the proportion of triptan prescriptions showed a marked decreasing trend. This may be related to the Chinese health administration and hospital prescription cost control, where some high-priced triptan prescriptions were diverted to pharmacy purchases.

In our study, flunarizine was the most prescribed preventive medication, accounting for half of the preventive prescriptions with a lower drug expenditure. This may differ from the epidemiologic results of migraine prevention in countries such as the United States and the United Kingdom, where flunarizine is not yet available. Another noteworthy point is that, except for flunarizine, most of the other recommended preventive medications are off-label in China, which may be one of the reasons why flunarizine was chosen more frequently than other medications. Although there is evidence that flunarizine is effective in preventing migraines, it may increase the proportion of patients who discontinue treatment due to adverse events such as sedation and weight increase [23, 24]. A decrease in the use of flunarizine was observed during the study period, with a concomitant increase in the use of other evidence-based and guideline-recommended medications. The anticonvulsant topiramate showed an upward trend in prescribing and drug expenditures. Several high-quality studies have shown that topiramate is an effective preventive medicine for migraine [25, 26]. Limited data from comparative trials suggest that topiramate may have a modest advantage over valproate for episodic migraine prevention [25]. Adverse events associated with topiramate therapy include depression and weight loss, generally mild to moderate in severity [27, 28]. Its evidenced efficacy and favorable safety profile may be the major reasons for its increasing use.

The drug class with the largest expenditure on preventive medication was antidepressants (34.18%), while the expenditure showed a significant downward trend, which may benefit from China’s centralized drug procurement policy. Flupentixol/melitracen, rather than the recommended drug amitriptyline and venlafaxine, was the most commonly prescribed antidepressant for migraine prevention. Amitriptyline and venlafaxine are the most well-evidenced antidepressants and have been proven to have a significant migraine preventive effect, especially when migraine coexists with tension-type headaches, depression, or sleep disorders [29, 30]. Flupentixol/melitracen, a mixture of a tricyclic antidepressant and a classical antipsychotic component, is reported to be associated with significant improvement in quality of life [31]. However, there is very limited evidence to support the use of flupentixol/melitracen for migraine prevention. Special concern should be raised for the safety and rational use of this medicine, for which there is weak clinical evidence. Gratefully, we have observed a gradual decline in the percentage of flupentixol/melitracen use, suggesting that the irrational choice of this drug is gradually improving.

During the study period from 2018 to 2022, the medications used for acute migraine and migraine prevention were traditional medicines. The newer groups of medicines that target 5HT1F receptor and calcitonin gene-related peptide (CGRP) molecule and receptor have been recently approved and marketed in various countries [28, 32]. Rimegepant, a CGRP antagonist, was approved for acute migraine treatment in China in 2024, and anti-CGRP(R) monoclonal antibodies, erenumab and galcanezumab, were approved for migraine prevention in China in 2023 and 2024. The availability of these new drugs may change the future of migraine treatment.

In most cases, monotherapy is recommended for acute migraine. Nonopioid analgesics are the first choice, followed by triptans; if these are insufficient, combined therapy is used. Our study revealed that 3.88% of all therapeutic prescriptions were combined therapies, most of which were triptan-based combinations. Combining triptans with NSAIDs appears to have a positive benefit in treating acute migraine pain, with the best-studied combination of triptans with NSAIDs. Several randomized placebo-controlled trials and meta-analyses revealed that the combination of sumatriptan and naproxen was well tolerated, with more effective headache relief than placebo or sumatriptan alone at 2 hours after dosing and a better sustained pain-free response than sumatriptan monotherapy and naproxen monotherapy [33]. The combination of triptans with acetylsalicylic acid or acetaminophen may be associated with slightly better clinical outcomes than triptans alone [34]. A total of 8.63% of the preventative prescriptions were combined therapies, half of which involved flunarizine-based combinations. There are fewer studies on combined therapy for migraine prevention. One study showed that flunarizine combined with duloxetine was effective in improving neuroelectrophysiological indices, reducing inflammation, and alleviating depression and anxiety in chronic migraine patients with depression and anxiety [35]. Due to the lack of epidemiologic data, we were unable to assess the appropriateness of the combination therapy in our study.

In addition, avoiding medication overuse is an issue that cannot be ignored during acute migraine treatment. Medication overuse can lead to the development of MOH, which transforms episodic migraine into a chronic headache disorder [36, 37]. It also produced higher per-prescription costs. In the present study, 69.21% of opioid prescriptions, 38.53% of caffeine-containing agent prescriptions, 26.61% of NSAID prescriptions, 13.97% of acetaminophen prescriptions, and 6.03% of triptan prescriptions were considered to be written overuse. Suppression of medication overuse has been proven to be an effective method to reduce headache crisis [38]. There is a need for government and medical institutions to limit the prescribing of opioids, which are considered to have the highest risk of MOH [39]. The quantity and course of analgesic medications should be limited to 10 days per prescription for caffeine-containing agents and triptans and 15 days per prescription for NSAIDs and acetaminophen [36]. Preventive therapies should be used as the mainstay treatment for patients with frequent headaches.

This study also has several limitations. Our analysis represents only the results of outpatient prescriptions for migraine and does not represent the overall use of antimigraine medications, as some patients obtain medications outside of hospital pharmacies. Our analysis was based on prescription data, and the appropriateness of the prescriptions, the duration of therapy, clinical efficacy, and safety could not be evaluated. Many anti-migraine medications have other indications; despite our screening, there may still be an overestimation of certain anti-migraine medications. Finally, the migraine prescription data were extracted from hospitals in major cities of China, which may cause sampling bias.

## Conclusions

Analgesics were commonly prescribed therapeutic medications, and flunarizine was the most prescribed preventive medicine. The use of migraine treatment has gradually converged toward evidence-based and guideline-recommended treatment. Opioid prescriptions, weak evidence-based antidepressant use, and written medication overuse prescriptions need to be urgently corrected. This study provides a basis for the future management of migraine treatment by the government and medical institutions.

### Supplementary information

**Supplementary Material 1.**

## Data Availability

Data is provided within the manuscript or supplementary information files.

## Abbreviations

GBD: Global burden of diseases
ICD: International statistical classification of diseases and related health problems
NSAIDs: Nonsteroidal anti-inflammatory drugs
CPMs: Chinese patent medicines
CCBs: Calcium channel blockers
CCMs: Calcium channel modulators
CNY: Chinese yuan
MOH: Medication overuse headache
TCM: Traditional chinese medicine
CGRP: Calcitonin gene-related peptide
