# Supplementary material for: Trends and prescribing patterns of antimigraine medicines in nine major cities in China from 2018 to 2022: a retrospective prescription analysis
Source: J Headache Pain. 2024 Apr 23;25(1):62. doi: 10.1186/s10194-024-01775-6 (PMC11036710; doi:10.1186/s10194-024-01775-6)
Supplement: Supplementary file 1 — Supplementary Material 1. [file 10194_2024_1775_MOESM1_ESM.docx]

**Supplementary Table 1. Antimigraine medications within the therapeutic or preventive medication category**

| **Drug Classification** | **Drug name** |
| --- | --- |
| NSAIDs | Ibuprofen (ibuprofen, dexibuprofen, ibuprofen arginine), Loxoprofen, Celecoxib, Diclofenac, Indomethacin, Naproxen, Meloxicam, Imrecoxib, Nimesulide, Etoricoxib, Lornoxicam, Ketorolac, Piroxicam, Lysine acetylsalicylate, Aspirin (lysine acetylsalicylate, aspirin) |
| Caffeine-contained agents | Acetaminophen/caffeine, Aminopyrine/caffeine, Acetaminophen/propylantipyrine/caffeine, Aspirin/acetaminophen/caffeine, Aminopyrine/phenacetin/phenobarbital/caffeine, |
| Opioids | Codeine/ibuprofen, Dihydrocodeine/acetaminophen, Oxycodone/acetaminophen, Tramadol, Codeine/acetaminophen, Tramadol/ acetaminophen |
| Acetaminophen | Acetaminophen |
| CPMs | Rotundine, Bulleyaconitine, Fengtongning (sinomenone), Anluotong (Marasmiellus androsaceus extract) |
| Triptans (orally) | Rizatriptan, Zolmitriptan, Sumatriptan |
| Ergotamine | Dihydroergotoxine |
| Antiemetics | Metoclopramide, Chlorpromazine, Promethazine, Domperidone, Mosapride, Itopride, Tropisetron, Diphenhydramine |
| CCBs | Flunarizine, Lomerizine |
| Antidepressants | Flupentixol/melitracen, Amitriptyline, Escitalopram, Duloxetine, Venlafaxine, Doxepin, Paroxetine, Trazodone, Fluvoxamine, Citalopram, Vortioxetine, Mirtazapine |
| Anticonvulsants | Topiramate, Valproate, Oxcarbazepin, Phenytoin, Carbamazepine, Lamotrigine, Levetiracetam |
| CCMs | Gabapentin, Pregabalin |
| Beta blockers | Metoprolol, Propranolol |
| Other agents | Candesartan, Botulinum toxin A, Riboflavin |
